# Apoptotic and Autophagic Effects of *Sesbania grandiflora* Flowers in Human Leukemic Cells

**DOI:** 10.1371/journal.pone.0071672

**Published:** 2013-08-13

**Authors:** Rajneeta Roy, Deepak Kumar, Biswajit Chakraborty, Chinmay Chowdhury, Padma Das

**Affiliations:** 1 Cancer Biology and Inflammatory Disorder Division, CSIR-Indian Institute of Chemical Biology, Kolkata, West Bengal, India; 2 Chemistry Division, CSIR-Indian Institute of Chemical Biology, Kolkata, West Bengal, India; University of Pecs Medical School, Hungary

## Abstract

**Background:**

Identification of cytotoxic compounds that induce apoptosis has been the mainstay of anti-cancer therapeutics for several decades. In recent years, focus has shifted to inducing multiple modes of cell death coupled with reduced systemic toxicity. The plant *Sesbania grandiflora* is widely used in Indian traditional medicine for the treatment of a broad spectrum of diseases. This encouraged us to investigate into the anti-proliferative effect of a fraction (F2) isolated from *S. grandiflora* flowers in cancer cells and delineate the underlying involvement of apoptotic and autophagic pathways.

**Principal Findings:**

Using MTT based cell viability assay, we evaluated the cytotoxic potential of fraction F2. It was the most effective on U937 cells (IC_50_∶18.6 µg/ml). Inhibition of growth involved enhancement of Annexin V positivity. This was associated with elevated reactive oxygen species generation, measured by flow cytometry and reduced oxygen consumption – both effects being abrogated by anti-oxidant NAC. This caused stimulation of pro-apoptotic proteins and concomitant inhibition of anti-apoptotic protein expressions inducing mitochondrial depolarization, as measured by flow cytometry and release of cytochrome c. Interestingly, even with these molecular features of apoptosis, F2 was able to alter Atg protein levels and induce LC3 processing. This was accompanied by formation of autophagic vacuoles as revealed by fluorescence and transmission electron microscopy – confirming the occurrence of autophagy. Eventually, F2 triggered caspase cascade – executioners of programmed cell death and AIF translocation to nuclei. This culminated in cleavage of the DNA repair enzyme, poly (ADP-ribose) polymerase that caused DNA damage as proved by staining with Hoechst 33258 leading to cell death.

**Conclusions:**

The findings suggest fraction F2 triggers pro-oxidant activity and mediates its cytotoxicity in leukemic cells via apoptosis and autophagy. Thus, it merits consideration and further investigation as a therapeutic option for the treatment of leukemia.

## Introduction

Leukemia, the most common hemato-oncological disorder, is characterized by heterogeneous group of neoplasm arising from malignant transformation of haematopoietic cells [1]. Despite the increasing understanding of the prognosis of leukemia, there is still a strong need for novel and effective pharmacological strategies for intervention with this disease. Different combinational chemotherapies are available but not without difficulties like incidence of drug resistance coupled with adverse effects and high treatment costs. This sets out the need to explore alternative therapeutic agents [2].

It has been found that some medicinal plants are potential sources for chemical molecules having useful biological activity of great diversity. Ethnobotanical knowledge coupled with rationale-driven scientific research has formed an important facet of anti-cancer drug discovery because medicinal plants have a very long history of safe consumption, and bioactive compounds obtained from them are normally non-toxic or less toxic to humans [3].

*Sesbania grandiflora* (SG) a member of the family Papilionaceae and commonly known as “sesbania” and “agathi”, is an important source of dietary nutrients in Southeast Asian countries [4]. All parts of SG including preparations derived from the roots, bark, gum, leaves, flowers, and fruit are utilized for the treatment of a broad spectrum of diseases including leprosy, gout, rheumatism, tumor and liver disorders [5]. Roots are applied as poultice for application to inflammation and fever. The flowers and leaves are reportedly associated with anti-inflammatory, analgesic, antipyretic and anti-epileptic effects [6], [7]. Additionally, juices derived from its flowers have a special ability to improve vision and the crushed leaves are applied to sprains and bruises. Recently, anxiolytic [8], hepatoprotective [9], cardio protective [10], anti-urolithiatic [11] activities and chemo-preventive efficacy [12], [13] of the plant have been reported. However, so far no investigation has been carried out into the multiple modes of cell death caused by SG.

Growth inhibition and induction of programmed cell death are among the major objectives of anti-cancer therapies. Several types of cell death have been classified and defined by the Nomenclature Committee on Cell Death (NCCD), including apoptotic and autophagic cell death [14]. Apoptosis is characterized by cell shrinkage, DNA fragmentation, chromatin condensation, pyknotic nuclei and production of apoptotic bodies. In contrast, autophagy is an intracellular degradation system involving sequestration of cytoplasm and organelles into double-membrane vesicles that traffic the contents to lysosomes for degradation. Although apoptosis is adjudged to be the main mechanism underlying anti-tumor activity, it does not function alone to determine a cell’s fate [15]. Drug-induced autophagy is being increasingly implicated in modulating cell death responses. In some cases, activation of autophagy is a cellular survival strategy but persistent activation of autophagy can lead to cell death. This implies that autophagy leading to cell death or survival is circumstantial. Recent studies show that extensive overlap exists between apoptotic and autophagic modes of cell death. The two forms of cell death are shown to have common aspects and precede each other or even coexist, suggesting that both apoptosis and autophagy are important target mechanisms for novel therapeutic agents [16].

In this study, we report some promising results obtained from a fraction (F2) derived from flowers of SG in U937 human leukemic cells. F2 can induce apoptosis in U937 cells evidenced by phosphatidylserine exposure, decrease in mitochondrial membrane potential and altered levels of apoptotic proteins. Interestingly, it can also induce autophagy by activating Atg proteins, LC3 conversion and autophagosome formation. The fact that F2 initiates both autophagic cell death and apoptosis in U937 cells suggests that it has potential for anti-cancer therapy.

## Materials and Methods

### Ethics Statement

Blood samples were obtained following informed written consent from normal healthy volunteers. Ethical approvals for the study and consent procedure were obtained from Internal Review Board (*Ethical Committee on Human Subjects*) of CSIR-Indian Institute of Chemical Biology. All clinical investigations have been conducted according to the principles expressed in the Declaration of Helsinki.

### Materials

All chemicals were obtained from Sigma-Aldrich (St. Louis, Missouri, USA) except 3-(4,5-Dimethylthiazol-2-yl)-2,5-diphenyltetrazolium bromide (MTT) was purchased from USB Corporation (USA). Pen strep, RPMI 1640, DMEM, Heat inactivated Fetal Bovine Serum (FBS), 5, 5′, 6, 6′-tetrachloro-1,1′, 3, 3′-tetraethylbenzimidazolylcarbocyanine iodide (JC-1), and 5-(and-6)-chloromethyl-2′,7′-dichlorodihydrofluorescein diacetate (CM-H_2_DCFDA) were obtained from Invitrogen (Carlsbad, CA, USA). Z-Val-Ala-DL-Asp (methoxy)-fluoromethylketone (Z-VAD-FMK) was obtained from BD Biosciences (San Jose, CA, USA). Caspase-3, Caspase-8, Caspase-9 colorimetric assay kits and nuclear/cytosol, mitochondria/cytosol fractionation kits were procured from Biovision (Milpitas, CA, USA). The antibodies against Bcl-2, Bax, β-Actin, cytochrome c, Poly ADP ribose polymerase (PARP), Apoptosis Inducing Factor (AIF), Laminin, Alkaline phosphatase/Horseradish peroxidase conjugated secondary antibodies and enhanced chemiluminescence kit were purchased from Santa Cruz Biotechnology (Santa Cruz, CA, USA). The antibodies against Beclin-1, LC3, Atg 3, Atg 5, Atg 7, Atg 12 were procured from Cell Signaling Technology (Inc. Beverly, MA, USA).

### Cell Lines

Six human cell lines namely U937-leukemic monocytic lymphoma, THP1-acute monocytic leukemia, Raji-Burkitt’s lymphoma, HL60-promyelocytic leukemia, MCF-7-breast adenocarcinoma, HCT-15-colon carcinoma and NIH3T3-non cancerous mouse embryonic fibroblast cell line were obtained from National Centre for Cell Science, Pune, India. U937, THP1, Raji, HL60, MCF-7 and HCT-15 were maintained in RPMI-1640 medium (pH = 6.8), NIH3T3 in DMEM (pH = 6.8), supplemented with 10% FBS and antibiotics (50 IU/ml penicillin G and 50 µg/ml streptomycin). The cells were incubated at 37°C in a humidified incubator containing 5% CO_2_ and subcultured every 72 h using an inoculum of 5×10^5^ cells/ml. Cell viability (>95%) was confirmed by trypan blue exclusion.

### Isolation of Human Peripheral Blood Mononuclear Cells (PBMC)

PBMC were isolated from anticoagulated blood of five healthy donors by density gradient centrifugation on an equal volume of Ficoll-Hypaque (Histopaque-1077) at 400×g for 30 mins. PBMC were harvested from the interface, washed twice in phosphate buffered saline (PBS, 0.01 M, pH 7.4) and resuspended in RPMI-1640 medium supplemented with penicillin (50 IU/ml), streptomycin (50 µg/ml) and 10% FBS [17]. Cell viability was confirmed by trypan blue exclusion (>95%).

### Preparation of *Sesbania grandiflora* Extracts

Initially, 1.0 kg of the dried flowers of the *Sesbania grandiflora* was grounded and then extracted (cold) with methanol two times (3 L×2) followed by methanol-water (1∶1) (3 L×1). The combined extracts were evaporated to dryness, affording a syrupy residue. It was then fractionated using hexane (1 L×2), ethyl acetate (1.5 L×2) and n-butanol (1 L×2), respectively. After screening these fractions, the n-butanol fraction displayed highest activity and thus, it was considered as a activity guided fraction. Next, n-butanol extract was subjected to a column chromatography over silica gel (100–200 mesh) column (100×15 cm) and eluted with different solutions with increasing polarity (e.g., 1∶1 chloroform: petroleum ether (v/v), 80% chloroform-petroleum ether (v/v), chloroform, 0.5% methanol-chloroform (v/v), 2% methanol-chloroform (v/v), 5% methanol-chloroform (v/v), 10% methanol-chloroform (v/v), 20% methanol-chloroform (v/v) etc.) to afford a total of 36 fractions of 50 mL each. Column fractions were analyzed by TLC (silica gel 60 F254) and fractions with similar TLC pattern were combined to give four major fractions (F1, F2, F3 and F4). All of these fractions were screened for anti-cancer activities. However, F2 fraction eluted with 2% methanolic chloroform was found to be the most active one stimulating our interest for studies in details as discussed in this article.

### Cell Viability Assay

The cytotoxic activity of the solvent extracts (hexane, ethyl acetate, n-butanol) of SG flowers and fractions (F1, F2, F3, F4) of n-butanol extract was evaluated in U937, THP1, Raji, HL60, MCF-7, HCT-15, NIH3T3 cells and PBMC using MTT assay as described previously [18]. Briefly, cells (1.25–2.5×10^4^ cells/100 µl of RPMI 1640 medium/well) were seeded in 96-well tissue culture plates and incubated with hexane, ethyl acetate, n-butanol extracts (0–100 µg/ml) and F1, F2, F3, F4 fractions of n-butanol extract (0–50 µg/ml) for 48 h at 37°C, 5% CO_2_. The time-dependent nature of cytotoxicity of F2 in U937 cells was also evaluated by incubating with F2 for 0–72 h. Following treatment, cell viability was measured by adding 20 µl MTT (5 mg/ml in PBS) and incubated for 4 h at 37°C. Subsequently, 100 µl DMSO was added to each well, resultant optical densities were measured at 540 nm in an ELISA Reader (BIO RAD, CA, USA). Accordingly, the specific absorbance that represented formazan production was calculated by subtraction of background absorbance from total absorbance. The mean percentage viability was calculated as follows:
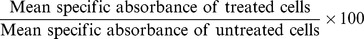



The results were expressed as IC_50_ values, i.e. the concentration that inhibited 50% of cell growth, which was enumerated by graphical extrapolation using Graph Pad Prism software (version 5, GraphPad Software Inc, San Diego, CA, USA). Each experiment was performed at least three times and in duplicate. PBMC (2.5×10^4^ cells/100 µl of RPMI 1640 medium/well) was incubated with F2 fraction (0–100 µg/ml; 48 h) and cell viability was similarly measured.

### Apoptosis Assay

Translocation of phosphatidylserine from the inner aspect to the outer leaflet of the plasma membrane occurs during apoptosis which can be ascertained by exploiting the high binding affinity of Annexin V, a Ca^+2^ dependent phospholipid binding protein to phosphatidylserine. In contrast, Propidium Iodide (PI), a non permeable stain has affinity towards nucleic acids and selectively enters necrotic or late apoptotic cells. To examine whether cell death occurred via apoptosis or necrosis, double staining was performed as described previously [19]. Therefore, co-staining of Annexin V and PI helps to discriminate between live cells (PI and Annexin V negative; lower left quadrant), cells in early apoptosis (Annexin V positive, PI negative; lower right quadrant), cells undergoing late apoptosis (both Annexin V and PI positive; upper right quadrant) or necrotic cells (PI positive, Annexin V negative; upper left quadrant). Briefly, U937 cells (2.5×10^5^/ml) were incubated with or without F2 (IC_50_∶18.6 µg/ml) for 12 h, 24 h and 48 h at 37°C, 5% CO_2_. Cells were then washed twice in PBS and resuspended in Annexin V binding buffer (10 mM HEPES, 140 mM NaCl, 2.5 mM CaCl_2_; pH 7.4). Annexin V-FITC was then added according to the manufacturer’s instructions and incubated for 15 min under dark conditions at 25°C. PI (0.1 µg/ml) was added just prior to acquisition. Data was acquired using a FACS Aria flow cytometer (Becton Dickinson, USA) at an excitation wavelength of 488 nm and an emission wavelength of 530 nm and analyzed with BD FACS Diva software (Becton Dickinson, USA).

### Measurement of Intracellular Reactive Oxygen Species (ROS)

Mitochondria frequently increase ROS generation during apoptosis that results in oxidative stress and cellular damage. To examine the effect of F2 fraction on generation of ROS, CM-H_2_DCFDA – lipid soluble, membrane permeable non-fluorescent reduced derivative of 2,7-dichlorofluorescein was used based on the evidence that the acetate groups of CM-H_2_DCFDA are removed by esterase cleavage intracellularly to produce hydrophilic, non-fluorescent dye Dichlorodihydrofluorescein (DCFH_2_) which is subsequently oxidized by ROS to form a highly fluorescent product Dichlorofluorescein (DCF). Thus, the fluorescence generated is directly proportional to the quantum of ROS generated [20]. The effect of IC_50_ concentration of F2 (18.6 µg/ml for U937, 20.9 µg/ml for THP1, 24.6 µg/ml for HL60, 26.1 µg/ml for Raji) on generation of ROS (0–2 h) was measured in cells (2.5×10^5^/ml). Also, the effect of varying dose on ROS generation was investigated in U937 cells by incubating them with 0–25 µg/ml F2 for 30 min. To corroborate the non-toxic effect of F2 in healthy cells, PBMC (2.5×10^5^/ml) were incubated with F2 (18.6 µg/ml; 0–2 h). After treatment, cells were washed with PBS (2000×g; 5 min), resuspended in PBS and then incubated with CM-H_2_DCFDA (5 µM in PBS) for 30 min at 37°C. Subsequently, cells were again washed and resuspended in PBS. DCF fluorescence was determined by flow cytometry at an excitation wavelength of 488 nm and an emission wavelength of 530 nm. To confirm the elevated levels of ROS induced by F2 fraction, for the inhibition of ROS generation, cells were pre-incubated with N-Acetyl cysteine (NAC), an established anti-oxidant (2.5 mM) for 3 h before treatment with F2. Mean fluorescence intensities (MFI) were obtained using the FACS Diva software. To determine the role of ROS in F2 mediated action, cells pre-incubated with NAC (2.5 mM; 3 h) were treated with F2 and analyzed for cell viability assay and apoptosis assay.

### Measurement of Oxygen Consumption in U937 Cells

Mitochondria are the hub of regulation of cellular respiration and energy homeostasis since they consume about 90% of cellular oxygen. Oxygen consumption by cells was measured as an indication of the mitochondrial respiration activity [21]. Control and F2 fraction treated (18.6 µg/ml; 30 min) cells (2.5×10^5^/ml) were washed and resuspended in 1 ml of fresh RPMI 1640 medium and then placed in the sealed respiration chamber equipped with a thermostat control and a micro-stirring device (Oxytherm, Hansatech Instrument, England). Oxygen consumption was measured polarographically at 37°C with the Clark-type oxygen electrode disc, using the conditions recommended by the manufacturer. The oxygen content in the suspension medium was constantly monitored, and the signals were integrated using the software supplied by the manufacturer. The oxygen content in the starting medium was normalized assuming an O_2_ concentration of 220 µM in air-saturated medium at 37°C. To explore the effect of NAC on oxygen consumption, cells were pre-incubated with NAC (2.5 mM; 3 h) followed by treatment with F2 (18.6 µg/ml; 30 min). Respiration rate is expressed as nanomoles of O_2_ consumed as a function of time (nmoles/L/min).

### Detection of Mitochondrial Membrane Potential

The mitochondria are attractive targets for cancer chemotherapy since its impairment renders cells non viable. The loss of mitochondrial potential is one of the indicators of apoptosis. The mitochondrial transmembrane electrochemical gradient (ΔΨ_m_) was measured using mitochondrial potential sensor JC-1, a cell permeable, cationic and lipophilic dye. In viable cells, it freely crosses the mitochondrial membrane and forms J-aggregates which fluoresce red. In apoptotic cells, decrease in mitochondrial membrane potential prevents JC-1 from entering the mitochondria and remains as monomers in the cytosol that emits a predominantly green fluorescence [22]. Therefore, the ratio of J-aggregates/monomers functions as an effective indicator of mitochondrial transmembrane potential and helps distinguish apoptotic cells from their healthy counterparts. Briefly, U937 cells (2.5×10^5^/ml) were incubated with F2 (18.6 µg/ml) for 6, 12, 24, and 48 h at 37°C, 5% CO_2_. The cells were then washed with PBS, incubated with JC-1 (7.5 µM in PBS) under dark conditions for 15 min at 20–25°C. Cells were acquired using FACS and analyzed using FACS Diva software. U937 cells treated with H_2_O_2_ (30 mM; 37°C; 15 min), illustrative of cells with depolarized mitochondrial membrane potential served to set the quadrants.

### Preparation of Cytosolic and Mitochondrial Extracts

Mitochondrial and cytosolic proteins were isolated using Mitochondrial Fractionation Kit according to the manufacturer’s instructions. Briefly, control and F2 fraction treated (18.6 µg/ml; 0–48 h) U937 cells were washed with ice-cold PBS and the supernatant was discarded. Pellet was resuspended in Cytosol Extraction Buffer [containing Dithiothreitol (DTT) and protease inhibitors] and homogenized. The homogenate was centrifuged at 700×g for 10 minutes at 4°C. The supernatant was collected and further centrifuged at 10,000×g for 30 minutes at 4°C. The supernatant was collected as the cytosolic fraction. The pellet containing the mitochondria was resuspended in mitochondria extraction buffer (containing DTT and protease inhibitors) and stored at −80°C until further use.

### Preparation of Cytosolic and Nuclear Fractions

To determine AIF translocation to nuclei, nuclear and cytosolic protein extractions were performed using Nuclear Fractionation Kit according to the manufacturer’s instructions. Briefly, control and F2 fraction treated (18.6 µg/ml; 0–48 h) U937 cells were washed with ice-cold PBS and the supernatant was carefully discarded. Cells were vortexed with ice-cold cytoplasmic protein extraction buffer A (containing DTT and protease inhibitors) for 15 sec followed by homogenisation. Cytoplasmic protein extraction buffer B was then added, and cell lysates were vortexed for 5 seconds, incubated on ice for 1 minute. This was centrifuged at 16,000×g for 5 min at 4°C to obtain supernatant (cytoplasmic proteins). Pellets were then re-suspended in nuclear protein extraction buffer (containing DTT and protease inhibitors) followed by being vortexed at 4°C for 10 min (20 sec pulse, followed by 2 min interval). Cell lysates were further centrifuged at 16,000×g for 10 min at 4°C, and supernatant was stored as nuclear protein extracts at −80°C until use.

### Western Blotting Analysis

Control and F2 treated (18.6 µg/ml; 0–48 h) cells were lysed in lysis buffer (50 mM Tris–HCl pH 7.4, 150 mM NaCl, 1 mM EDTA, 1 mM EGTA, 1µg/ml protease inhibitor cocktail, 5 mM PMSF and 1 mM DTT containing 1% Triton X-100), sonicated and centrifuged for 10 min at 4°C at 10,000×g and protein concentration estimated. Electrophoretic separations (50 µg protein/lane) were carried out on 10% SDS-polyacrylamide gel electrophoresis, and electrotransferred onto a PVDF membrane. Blots were blocked for 1 h at 37°C in 20 mM Tris-HCl, pH 7.4, 150 mM NaCl, 0.02% Tween 20 (TBST) containing 5% skimmed milk and probed using 1∶2000 dilution of appropriate antibodies (Bax, Bcl-2, β-Actin, PARP, cytochrome c, AIF, Laminin, Beclin-1, Atg 3, Atg 5, Atg 7, Atg 12) by incubating overnight at 4°C. The membranes were washed thrice with TBST, incubated with alkaline phosphatase/Horseradish peroxidase conjugated secondary antibody and the bands visualized using a 5-bromo-4-chloro-3-indolyl phosphate/nitro blue tetrazolium substrate or enhanced chemiluminescence kit.

### Caspase Activity Assay

Caspases, a family of cysteine proteases have emerged as key enzymes in the regulation of apoptotic pathway. Activation of caspases leads to the classical form of apoptotic pathway [23]. Measurement of caspase activation was based on the ability of the active caspase to cleave the chromophore from the enzyme substrate. The enzymatic activity of caspase −8, −9, −3 was assayed in cell lysates (100 µg protein in 50 µl lysis buffer) using colorimetric assay kits as per the manufacturer’s instructions. Briefly, control and F2 fraction treated (18.6 µg/ml; 0–48 h) cells (2.5×10^5^/ml) were washed with ice cold PBS, cell lysates were prepared and subsequently protein concentration was estimated. Lysates were combined with reaction buffer and incubated with specific colorimetric peptide substrates (Ac-IETD-pNA for Caspase-8, Ac-LEHD-pNA for Caspase-9, Ac-DEVD-pNA for Caspase-3; 4 mM, 5 µl) at 37°C for 6 h. The emission of paranitroanilide (pNA) was measured at 405 nm in an ELISA reader every 30 minutes for 6 h. To establish whether caspase activation contributes to F2 induced cytotoxicity, U937 cells (2.5×10^4^/100 µl of RPMI 1640 medium/well) were pre-incubated with pan caspase inhibitor Z-VAD-FMK (20 µM; 4 h) followed by treatment with F2 (0–100 µg/ml; 48 h) and cell viability was evaluated by MTT assay as described above. Also, in order to determine the effect of Z-VAD-FMK on F2 induced apoptosis, cells pre-incubated with Z-VAD-FMK (20 µM; 4 h) were treated with F2 (18.6 µg/ml; 48 h) and analysed by apoptosis assay.

### Cellular and Nuclear Morphology Analysis

Cell death culminates in nuclear condensation of chromatin and DNA degradation [24]. Nuclear morphology was analyzed by confocal laser scanning microscope, after staining with Hoechst 33258 in U937 cells. Control and F2 (18.6 µg/ml; 0–48 h) treated U937 (2.5×10^5^/ml) cells were washed in ice cold PBS and stained with Hoechst 33258 (10 µg/ml) [19]. The cells were mounted on Poly L-Lysine coated slides and analyzed in a laser scanning confocal microscope (Leica TCS SP2 System Leica Microsystem, Heidelberg, Germany, 100X). At least 20 randomly selected microscopic fields were observed per sample.

### Conversion of LC3

LC3 protein may exist in two forms – LC3-I and LC3-II. The conversion of microtubule-associated protein LC3-I to its membrane associated form LC3-II has been used as a marker of autophagy [25]. The conversion of LC3-I to LC3-II was observed using immunoblotting. Whole cell lysates were prepared from control and F2 treated (18.6 µg/ml; 0–48 h) U937 cells, protein concentration estimated and western blotting analysis done as described above. Densitometric quantification of signals was done using Imaging Densitometer (BIO RAD, CA, USA).

### Detection of Acidic Vesicular Organelles

Acidic vesicular organelles (AVO) were detected by acridine orange (AO) staining. AO is a weak base that traverses freely across biological membranes in an uncharged state characterized by green fluorescence. Its protonated form accumulates as aggregates in acidic compartments characterized by red fluorescence [26]. This differential staining helps identify cells with AVO. Briefly, control and F2 fraction (18.6 µg/ml; 0–48 h) treated U937 cells (2.5×10^5^/ml) were washed in PBS and were stained with AO (1 µg/ml) for 15 min at 25°C [27]. Cells were resuspended in PBS and observed under a fluorescence microscope at an excitation of 488 nm and emission of 530 nm and 650 nm. To confirm the role of autophagy in F2 fraction mediated cytotoxicity, U937 cells were pre-incubated with a non-toxic concentration of autophagy inhibitor 3-Methyl adenine (3-MA; 10 mM for 4 h) followed by treatment with F2 (0–100 µg/ml; 48 h). Cell viability was assessed by MTT assay as described before. Furthermore, cells pre-incubated with 3-MA (10 mM; 4 h) were treated with F2 (18.6 µg/ml; 48 h) and analyzed by apoptosis assay. In another set of experiment, cells (2.5×10^4^/100 µL of RPMI 1640 medium/well) were pre-incubated with both Z-VAD-FMK (20 µM) and 3-MA (10 mM) for 4 h, then treated with F2 (0–100 µg/ml; 48 h) and cell viability was similarly evaluated.

### Transmission Electron Microscopy

Formation of autophagosomes and autolysosomes is a characteristic hallmark of autophagic cells which is best visualized using transmission electron microscopy. Control and F2 treated (18.6 µg/ml; 0–48 h) U937 cells (2.5×10^5^/ml) were fixed in 2.5% glutaraldehyde and 2% paraformaldehyde in 0.1 M phosphate buffer (pH 7.4) for 1 h at 4°C. After being rinsed in PBS, cells were postfixed in osmium tetroxide (1%) for 2 h, dehydrated in graded acetone and embedded in araldite CY212. Semithin sections were cut, stained with 0.5% toluidine blue (5 min), and examined under a light microscope (Olympus, 60 X). Ultrathin sections were stained with 2% uranyl acetate and Reynold’s lead citrate, and observed with a transmission electron microscope (Technai G2) [28].

### Statistical Analysis

The statistically significant differences between control and drug-treated cells were calculated using one way ANOVA. Multiple comparisons were made between different treatments (analysis of variance) using Graph Pad Prism Software (version 5, GraphPad Software Inc, San Diego, CA, USA) and an error protecting the multiple comparison procedure, namely Tukey’s multiple comparison test. p<0.05 was considered as statistically significant. All data were expressed as mean± SEM/SD.

## Results

### *Sesbania grandiflora* Extract Exhibits Anti-proliferative Activity

**Figure 1:**
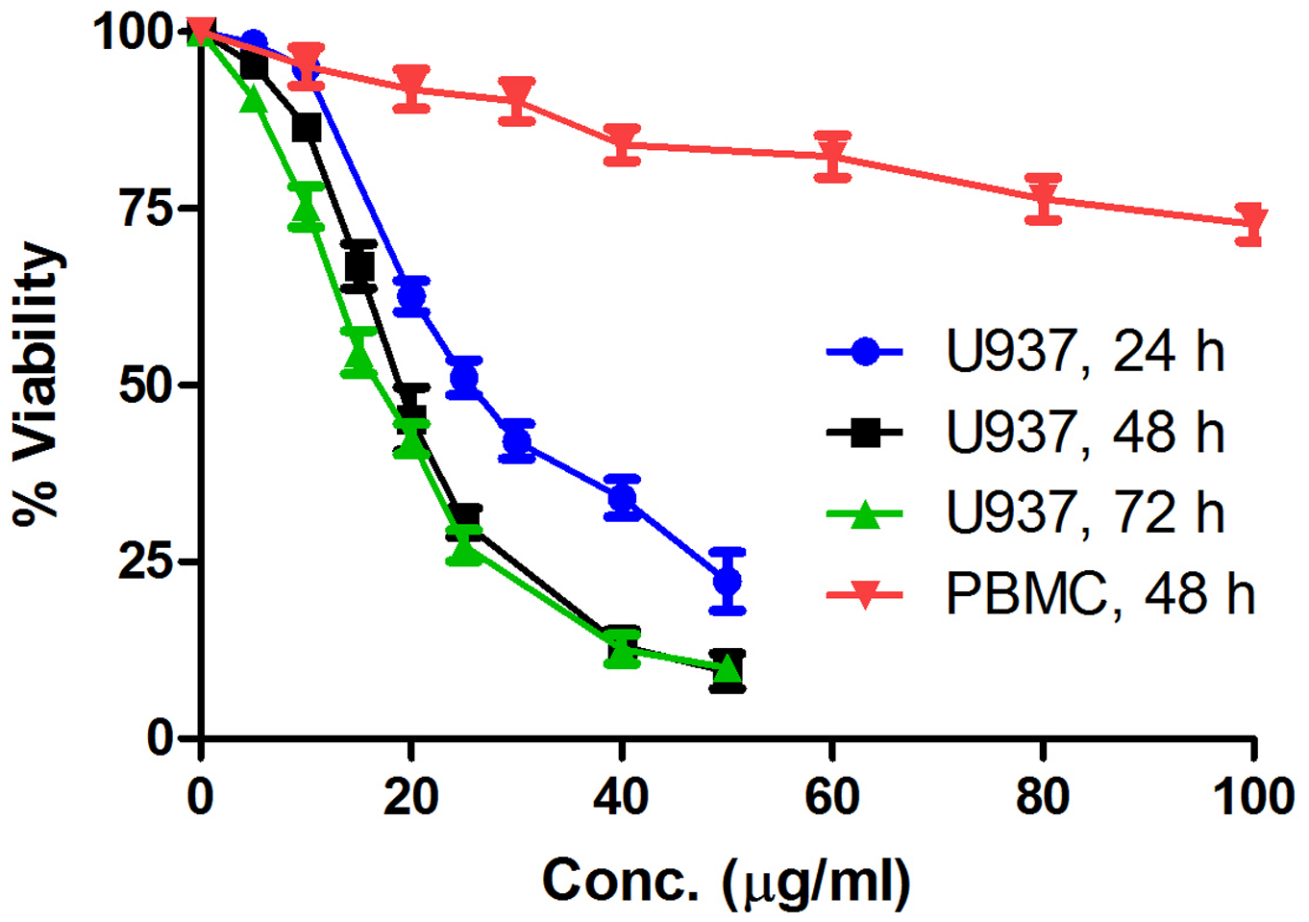
Dose response curve of U937 cells and PBMC after treatment with F2. Log phase U937 cells and PBMC (2.5×10^4^/100 µl of RPMI 1640 medium/well) were treated with F2 fraction of n-butanol extract [(0–50 µg/ml; 24, 48, 72 h for U937), and (0–100 µg/ml; 48 h for PBMC)] and cell viability was measured by MTT assay as described in Materials and methods. Each data point represents the Mean ± SEM of at least three independent experiments in duplicate.

Inhibition of cell proliferation was tested by MTT assay following treatment of cells with hexane, ethylacetate and n-butanol extracts. n-butanol extract was the most effective one and exhibited IC_50_ values of 30 µg/ml and 40 µg/ml respectively in U937 and THP1 cells (Table 1). When F1, F2, F3 and F4 fractions of n-butanol extract were tested for their cytotoxic potential in U937, THP1, HL60, Raji, MCF-7, HCT-15, NIH3T3 cells, and human PBMC, F2 was found to be the most potent fraction (Table 2); its IC_50_ values are 18.6 µg/ml in U937, 20.9 µg/ml in THP1, 24.6 µg/ml in HL60, 26.1 µg/ml in Raji and >50.0 µg/ml in MCF-7, HCT-15. F2 exhibited IC_50_ of 42.5 µg/ml in NIH3T3- mouse embryonic fibroblast cells. Also, F2 was unable to inhibit proliferation of human PBMC even at a concentration of 100 µg/ml. The results indicate that F2 does not confer any toxicity to healthy normal cells. F2 caused a marked growth inhibition and decrease in cell viability in dose and time dependent manner in U937 cells. After 24 h of treatment, the inhibitory concentration of F2 on the viability of U937 cells reached 25.6±1.3 µg/ml (Mean IC_50_±SEM). When the incubation time was prolonged to 48 h and 72 h, the cell growth inhibitory concentration decreased to 18.6±3.5 µg/ml and 16.9±2.0 µg/ml respectively (Fig. 1). Based on this, the F2 concentration of 18.6 µg/ml has been chosen for subsequent studies in U937. DMSO (0.1%), representative of the highest concentration used (100 µg/ml) showed no effect on cell viability, confirming its biological inertness.

**Table 1:** Cytotoxicity of extracts of flowers of *Sesbania grandiflora*.

Extract	Mean cell growth inhibition IC_50_ (µg/ml) ± SEM					
	U937	THP1	HL60	Raji	MCF-7	HCT-15
Hexane	>100.0	>100.0	>100.0	>100.0	>100.0	>100.0
Ethylacetate	>100.0	>100.0	>100.0	>100.0	>100.0	>100.0
n-butanol	30.0	40.0	>100.0	>100.0	>100.0	>100.0

Log phase cells (1.25–2.5×10^4^/100 µl of RPMI 1640 medium/well) were treated with hexane, ethyl acetate, n-butanol extracts of *Sesbania grandiflora* (0–100 µg/ml) for 48 h, and cell viability was measured by MTT assay as described in Materials and methods. Each IC_50_ (Mean ± SEM) has been derived from at least three experiments in duplicate.

**Table 2:** Cytotoxicity of fractions of n-butanol extract of flowers of *Sesbania grandiflora.*

Fraction	Mean cell growth inhibition IC_50_ (µg/ml) ± SEM							
	U937	THP1	HL60	Raji	MCF-7	HCT-15	NIH3T3	PBMC
F1	>50.0	>50.0	>50.0	>50.0	>50.0	>50.0	ND	ND
F2	18.6±3.5	20.9±1.8	24.6±2.3	26.1±2.8	>50.0	>50.0	42.5±0.9	>100.0
F3	>50.0	>50.0	>50.0	>50.0	>50.0	>50.0	ND	ND
F4	>50.0	>50.0	>50.0	>50.0	>50.0	>50.0	ND	ND

Log phase cells (1.25–2.5×10^4^/100 µl of RPMI 1640 medium/well) were treated with fractions (F1, F2, F3, F4) of n-butanol extract of *Sesbania grandiflora* (0–50 µg/ml) for 48 h, and cell viability was measured by MTT assay as described in Materials and methods. Each IC_50_ (Mean ± SEM) has been derived from at least three experiments in duplicate.

### F2 Causes Externalization of Phosphatidylserine

**Figure 2:**
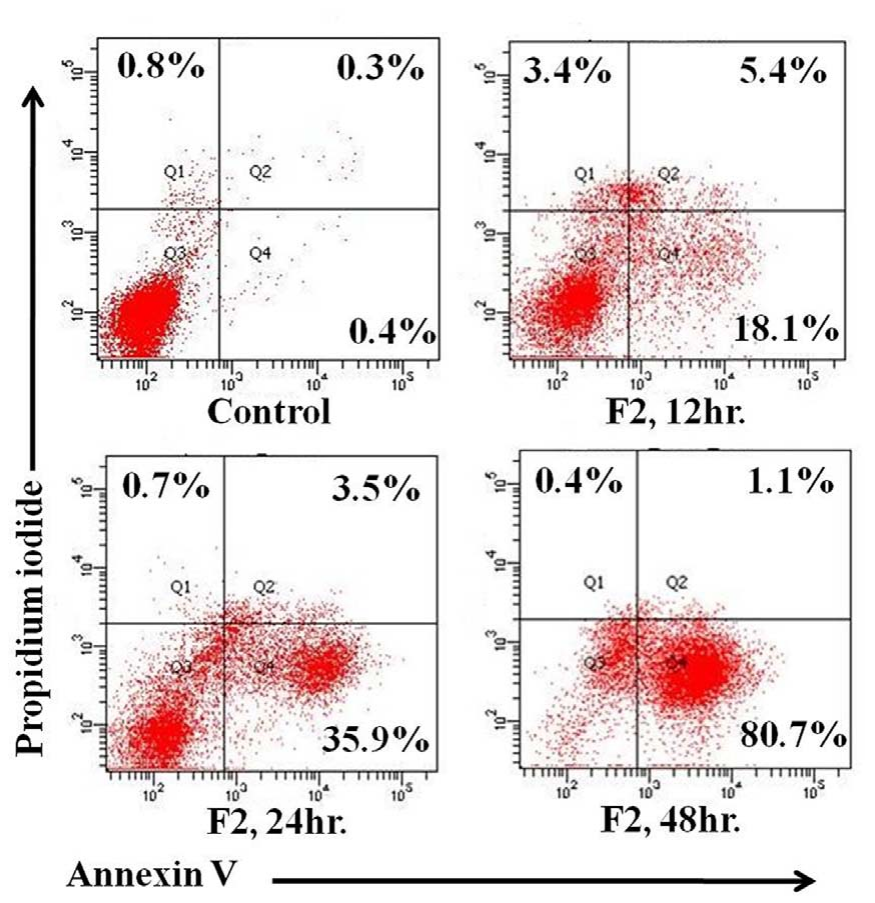
Induction of apoptosis by F2. Control and F2 treated (18.6 µg/ml; 12 h, 24 h, 48 h) U937 cells (2.5×10^5^/ml) were co-stained with PI and Annexin-V FITC followed by analysis using flow cytometry as described in Materials and methods. The figure is a representative profile of at least three experiments.

The appearance of phosphatidylserine on the external surface of plasma membrane serves as a marker for apoptosis. Annexin V-FITC and PI double-labeling was used for the detection of phosphatidylserine externalization [19]. Annexin V binding was detectable as early as 12 h (23.5±1.1%) after treatment, compared to control (0.7±1.6%, Fig. 2). After 24 h and 48 h of F2 exposure, 39.4±1.4% and 81.8±0.9% of U937 cells, respectively, were Annexin V positive. The percentage of PI positive cells (upper left quadrant) at baseline was minimal and remained comparable at 12, 24 and 48 h, being 3.4±1.1%, 0.7±0.04% and 0.4±0.5%, respectively implying negligible occurrence of necrosis. Taken together, F2 fraction causes externalization of phosphatidylserine, indicative of apoptosis.

### F2 Triggers ROS Generation

**Figure 3:**
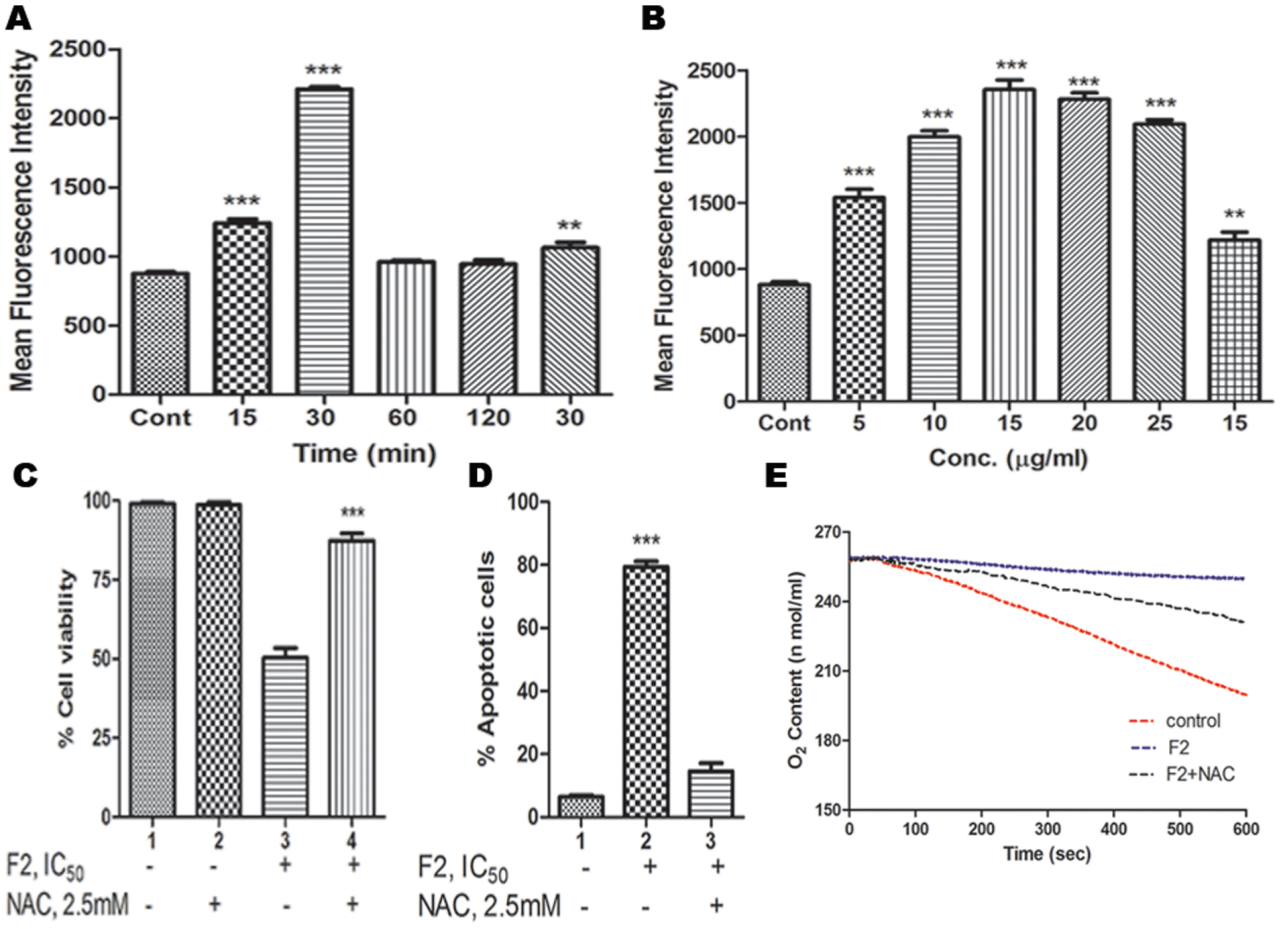
Effect of F2 on ROS generation and mitochondrial respiration. Increase in ROS levels. U937 (2.5×10^5^/ml) cells were treated with (A) IC_50_ concentration of F2 [18.6 µg/ml for 0–2 h (time-dependent) or (B) 0–25 µg/ml for 30 min (concentration dependent)]. After treatment, cells were washed and resuspended in PBS, incubated with CM-H_2_DCFDA (5 µM) for 30 min and the fluorescence was measured using flow cytometry as described in Materials and methods. For the inhibition of ROS generation, cells were pre-treated with NAC (2.5 mM) for 3 h before treatment with F2 (18.6 µg/ml; 30 min) and ROS was similarly quantified. Values are expressed as Mean Fluorescence Intensity ± SEM of three independent experiments (**p<0.01, ***p<0.001). (C) Effect of anti-oxidant on F2 induced cytotoxicity. Cells (2.5×10^4^/100 µl of RPMI 1640 medium/well) were pre-incubated with anti-oxidant NAC (2.5 mM; 3 h) followed by treatment with F2 (0–50 µg/ml; 48 h). Cell viability was evaluated by MTT assay as described in Materials and methods. Histograms represent percentage cell viability (Mean ± SEM) obtained at the IC_50_ concentration of F2 (18.6 µg/ml) and has been derived from at least three experiments in duplicate (***p<0.001 compared to only F2 treated cells). (D) Effect of anti-oxidant on F2 induced apoptosis. Cells were treated with F2 (18.6 µg/ml; 48 h) with or without pre-incubation using NAC (2.5 mM; 3 h). They were co-stained with Annexin-V FITC and PI followed by analysis using flow cytometry as described before. Histograms represent percentage apoptotic cells and have been derived from at least three experiments (***p<0.001 compared to control cells). (E) Inhibition of respiration. U937 cells were treated with F2 (18.6 µg/ml; 30 min) with or without pretreatment with NAC (2.5 mM; 3 h). Oxygen contents were monitored by using the Oxytherm system. Data shown are from one of the three experiments.

A main feature of cancer cells, when compared to normal ones, is a persistent pro-oxidative state that leads to an intrinsic oxidative stress. Persistent ROS stress may induce adaptive stress responses, enabling cancer cells to survive with high levels of ROS and maintain cellular viability. However, excessive ROS generation beyond a certain threshold level render cancer cells highly susceptible to chemotherapy induced death. Under such circumstances, ROS are known to function as the initial mediators of apoptosis [29]. To determine whether F2 fraction induced apoptosis is mediated by oxidative stress; the intracellular level of ROS was measured in U937, THP1, HL60, Raji cells, PBMC and the basal levels of ROS in terms of MFI were 874±13.6, 750.54±10.4, 800.47±12.4, 730.05±7.9, 450.1±5.9 respectively. We found that F2 (18.6 µg/ml for 0–2 h) was capable of producing ROS within 30 min in U937 cells (MFI = 2212.0±15.0, 2.5 fold increase compared to control, p<0.001; Fig. 3A). F2 also demonstrated a dose-dependent increase in levels of ROS in U937 cells (MFI being maximum and comparable at 15 µg/ml and 20 µg/ml, p<0.001; Fig. 3B). However, MFI of F2 induced ROS was 1000.8±7.9, 960.11±9.8, 1022±6.7, 510.1±7.4 respectively in THP1, HL60, Raji cells and PBMC – which are not significantly higher than their basal levels. This further potentiates the fact that F2 is most effective in U937 cells without causing stress to healthy cells. Pretreatment of U937 cells with anti-oxidant NAC (2.5 mM; 3 h) completely blocked the F2-induced increase in DCF fluorescence (MFI = 1063±38.2; 2.1 fold decrease compared to the maximum MFI of ROS generated). To exclude any autofluorescence generated by F2 fraction, cells were incubated with F2 and MFI was quantified. As no measurable increase in MFI was obtained, the observed increase in fluorescence was attributable to the ability of F2 to generate ROS in U937 cells.

To confirm whether the oxidative burst induced by F2 was a major contributory factor towards its anti-proliferative activity, U937 cells were pre-treated with NAC (2.5 mM; 3 h) followed by treatment with F2. The IC_50_ of F2 increased to 45.2 µg/ml from 18.6 µg/ml (Fig. 3C) and the percentage of apoptotic cells decreased to 9.2±1.9% (Fig. 3D), substantiating that induction of ROS is a key factor triggering the anti-proliferative activity of F2 in U937 cells.

### Inhibition of Oxygen Consumption by F2

Enhanced ROS generation is correlated to deregulated mitochondrial activity which gets reflected in subsided levels of cellular respiration [30]. Incubation of U937 cells with F2 (18.6 µg/ml) for 30 min led to a significant suppression of mitochondrial respiration, as evidenced by a substantial decrease in oxygen consumption (Fig. 3E). Rate of oxygen consumption in control cells was 5.78×nmoles/L/min which was subdued by 6.4 fold to 0.9×nmoles/L/min after treatment. To corroborate that F2 induced oxidative stress is playing a pivotal role in mediating its cytotoxicity, U937 cells were pre-incubated with anti-oxidant NAC (2.5 mM; 3 h) followed by treatment with F2. This substantially reversed the F2-mediated inhibition of respiration in U937 cells; the rate of oxygen consumption being 2.66×nmoles/L/min (3 fold increase compared to F2 treated cells) reiterating the critical contribution of ROS. These results also suggest that inhibition of mitochondrial respiration by F2 might be associated with alteration in ROS which is in concert with previous findings that ROS are inhibitors of mitochondrial activity [31].

### F2 Leads to the Induction of the Mitochondrial Pathway of Apoptosis

**Figure 4:**
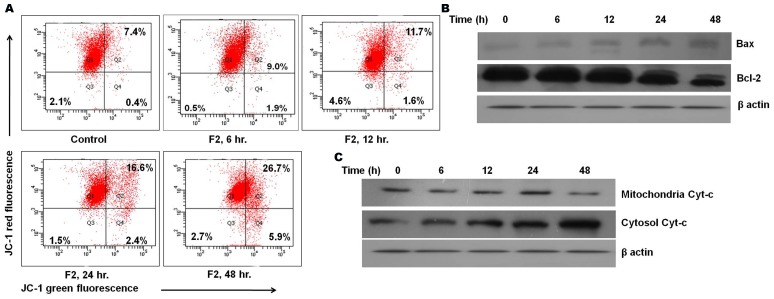
Involvement of mitochondrial pathway in F2 induced apoptosis. (A) Loss of mitochondrial membrane potential. U937 cells (2.5×10^5^/ml) were incubated with F2 (18.6 µg/ml for 6 h, 12 h, 24 h, 48 h). Cells were loaded with mitochondrial sensor dye JC-1 (7.5 µM; 15 min) as described in Materials and methods and analysis was revealed by the shift from red to green fluorescence in time dependent manner. Data is a representative of three different experiments. (B) The expression levels of pro- and anti-apoptotic proteins. Whole cell extracts were made from control and F2 treated U937 cells (2.5×10^5^/ml). Equal amounts of cell lysates were resolved by SDS-PAGE, transferred to PVDF membrane and probed with specific antibodies against Bcl-2, Bax. Analysis was confirmed with three different sets of experiments. (C) Effect of F2 on release of cytochrome c into cytosol. Cytoplasmic and mitochondrial fractions were prepared from control and F2 treated (18.6 µg/ml for 6 h, 12 h, 24 h, 48 h) U937 cells using mitochondria/cytosol fractionation kit and cytochrome c was analyzed by Western blotting with anti cytochrome c antibody. Data shown are from one of the three experiments. β-actin served as a loading control.

Since oxygen consumption is a surrogate indicator of mitochondrial bioenergetics, depleted cellular respiration coupled with increased ROS production may lead to mitochondrial depolarization [32]. Thus, loss of mitochondrial depolarization serves as one of the indicators of apoptosis. The mitochondrial membrane potential was measured by staining with JC-1, a lipophilic cationic fluorescent dye capable of selectively entering mitochondria and acting as a dual emission probe that reversibly changes color from green to red in concert with polarization of mitochondrial membrane [22]. The percentage of green fluorescence serves as an indicator of cells with depolarized mitochondria which may also be represented as the ratio of red/green fluorescence. A gradual time dependent change of red to green fluorescence was observed in U937 cells after treatment with F2 fraction (Fig. 4A). In control cells, the green fluorescence was 7.8% which increased to 10.9% at 6 h, 13.3% at 12 h, 19.0% at 24 h and 32.6% at 48 h of treatment. This was also reflected in the altered ratio of red/green fluorescence which in control cells was 11.8. Following the addition of F2, this ratio demonstrated a time-dependent decline (8.2, 6.5, 4.3, 2.1 at 6 h, 12 h, 24 h, 48 h respectively) suggesting the occurrence of depolarization of mitochondria by F2.

Studies suggest that alterations in cellular metabolism can lead to pro-apoptotic changes. By regulating mitochondrial membrane physiology, Bcl-2 proteins affect mitochondrial energy generation, and thus influence cellular bioenergetics [33]. Since a significant change in cellular respiration and ROS generation was observed, it may be envisaged that there may be alterations in levels of pro- and anti-apoptotic proteins of the Bcl-2 family. As evident from fig. 4B, a time-dependent increase in Bax levels, a pro-apoptotic protein was observed after F2 treatment accompanied by time-dependent decrease in expression of Bcl-2, an anti-apoptotic protein.

Furthermore, death-promoting members of the bcl-2 family, such as bax play key roles in the release of cytochrome c from mitochondria [2]. In order to delve into this, cytochrome c expression was analyzed in cytosolic and mitochondrial protein extracts of F2 treated cells. Our results show that F2 treatment promoted a time-dependent increase in the release of cytochrome c from mitochondria as evident from enhanced expression of cytochrome c in cytosolic fraction of treated cells (Fig. 4C). Based on these observations it may be suggested that F2 induced apoptosis of U937 cells is carried out through the mitochondrial pathway.

### F2 Induces Both Caspase-dependent and Caspase-independent Death in U937 Cells

**Figure 5:**
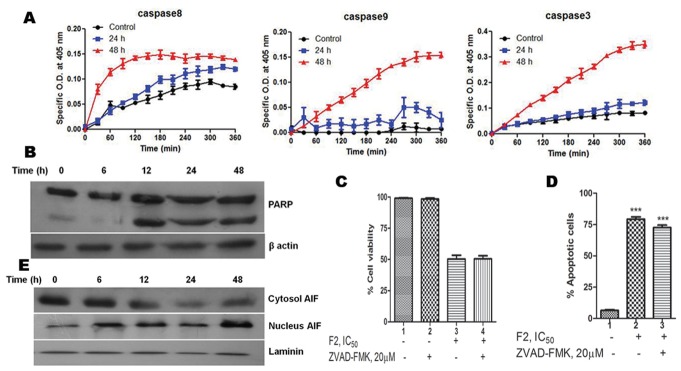
Involvement of caspase-dependent and caspase-independent modes of cell death. (A) Caspase activation. Activity of caspase-3, -8, -9 was measured in control and F2 (18.6 µg/ml) treated U937 cells (2.5×10^5^/ml) using colorimetric tetrapeptide substrates. Results are expressed as mean ± SEM from three independent experiments. (B) Cleavage of PARP by F2. Cell lysates were prepared and subjected to western blot analysis to check the cleavage of PARP. Data shown are from one of the three experiments. (C) Effect of pan caspase inhibitor on F2 induced cytotoxicity. Cells (2.5×10^4^/100 µl of RPMI 1640 medium/well) were pre-incubated with pan caspase inhibitor Z-VAD-FMK (20 µM; 4 h) followed by treatment with F2 (0–100 µg/ml; 48 h). Cell viability was evaluated by MTT assay. Histograms represent percentage cell viability (Mean ± SEM) obtained at the IC_50_ concentration of F2 (18.6 µg/ml) and has been derived from at least three experiments in duplicate. (D) Effect of pan caspase inhibitor on F2 induced apoptosis. Cells were treated with F2 (18.6 µg/ml; 48 h) with or without pre-incubation using Z-VAD-FMK (20 µM; 4 h). They were co-stained with Annexin V-FITC and PI followed by analysis using flow cytometry. Histograms represent percentage of apoptotic cells and have been derived from at least three experiments (***p<0.001 compared to control cells). (E) Translocation of AIF. Cytoplasmic and nuclear fractions were prepared from control and F2 treated U937 cells using nuclear/cytosol fractionation kit and translocation of AIF was analyzed by Western blotting. Analysis was confirmed with three different sets of extracts. Laminin served as a loading control.

Caspases have long been considered the pivotal executioners of programmed cell death. A change in the mitochondrial membrane permeability results in the release of apoptosis factors from mitochondria, which activate caspases [23]. Accordingly, activities of caspase-8, -9 and -3 was measured in cell lysates by quantitative detection of colorimetric tetrapeptide substrates. Caspase-8, -9 and -3 activities increased significantly after 48 h of F2 treatment (Fig. 5A). An exponential increase in the activity of caspase-9 (∼15 fold increase compared to control) and caspase-3 (∼4 fold increase compared to control) was observed up to 5 h after which the activity plateaued. However, for caspase-8 the exponential increase (∼2 fold increase compared to control) was observed up to 2 h. The activation of caspases was further corroborated by the specific cleavage of PARP (116 kDa), a known caspase substrate to yield an 85 kDa cleaved fragment (Fig. 5B). One of the most important functions of PARP is to help repair single-strand DNA nicks [3]. Thus, cleaved PARP - which is a useful marker for apoptosis leads to further cellular damage.

To confirm the role of caspase in F2 mediated cytotoxicity, U937 cells were pre-incubated with Z-VAD-FMK (20 µM; 4 h) followed by treatment with F2 for 48 h. The IC_50_ value remained unaltered at 18.6 µg/ml (Fig. 5C). Also, the percentage of apoptotic cells did not change significantly compared to only F2 treated cells (Fig. 5D). This implies that caspase activation occurs in F2 induced cell death. However, it does not exclusively determine the fate of F2 treated cells.

AIF release from mitochondria into cytosol followed by translocation into nucleus has been reported in context of caspase independent cell death in response to various death stimuli [34], [35]. Therefore, we next analyzed whether AIF is involved in F2 induced death of U937 cells. Results of western blot analysis revealed an increase of AIF expression in nuclear extract of F2 treated U937 cells implying the translocation of AIF to nucleus. Laminin served as the loading control (Fig. 5E). Collectively, this provides evidence for the ability of F2 to induce both caspase-dependent and -independent modes of cell death.

### F2 Alters Cellular and Nuclear Morphology

**Figure 6:**
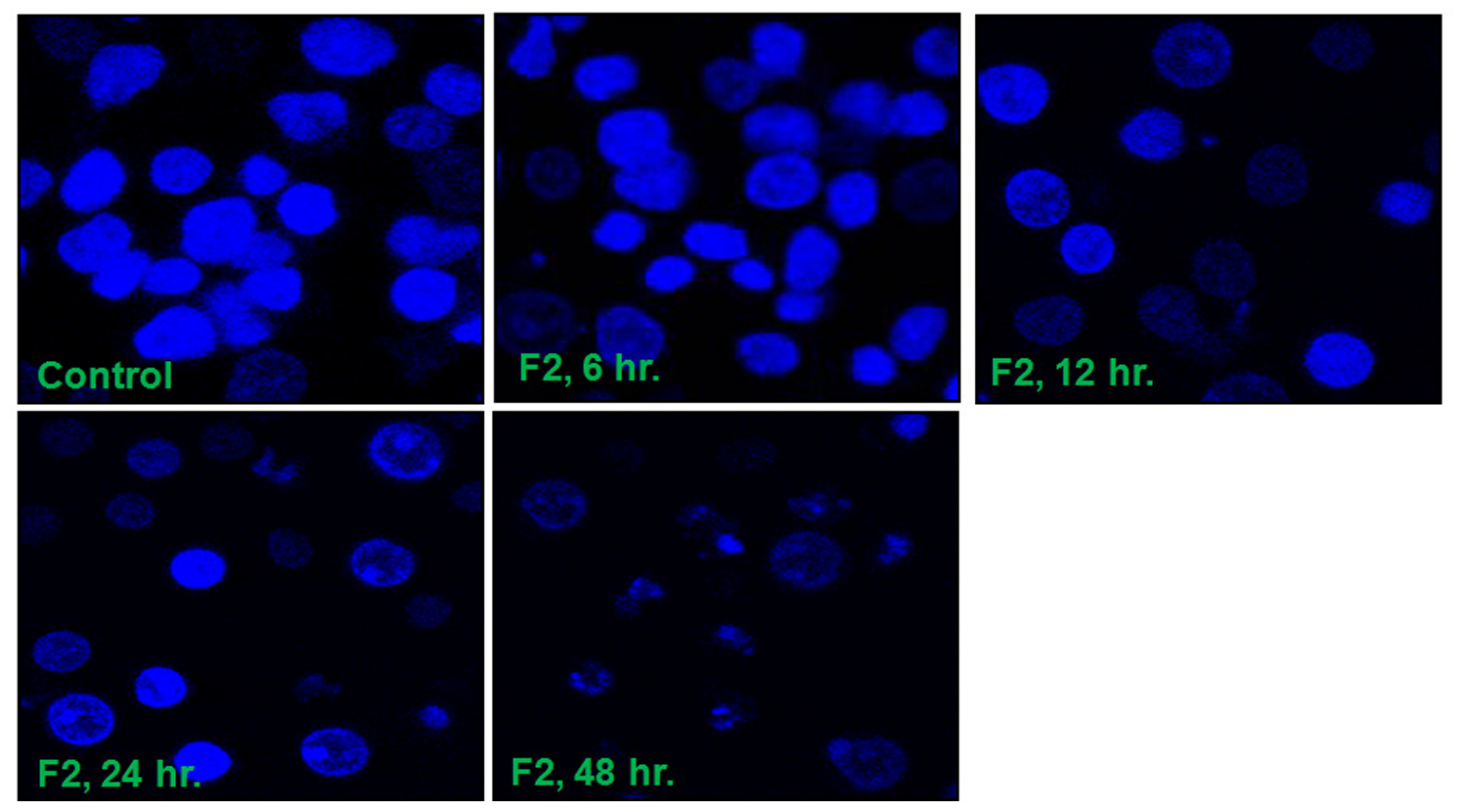
DNA fragmentation and morphological changes induced by F2. Control and F2 (18.6 µg/ml; 0–48 h) treated U937 cells (2.5×10^5^/ml) stained with Hoechst 33258 were observed under a Leica confocal microscope (100X). The figure is a representative profile of at least three experiments.

Caspases and AIF are actively involved in DNA fragmentation and chromatin condensation [35]. Fig. 6 shows the morphology of U937 cells treated with F2 and stained with Hoechst 33258. The confocal images showed that control cells possessed intact nuclei. In accordance with activation of caspases and translocation of AIF into nucleus, increased duration of treatment caused the formation of degraded nuclei, membrane blebbing and clear apoptotic bodies. This also supports the known phenotypic characteristics of apoptosis.

### F2 Alters Levels of Autophagic Proteins

**Figure 7:**
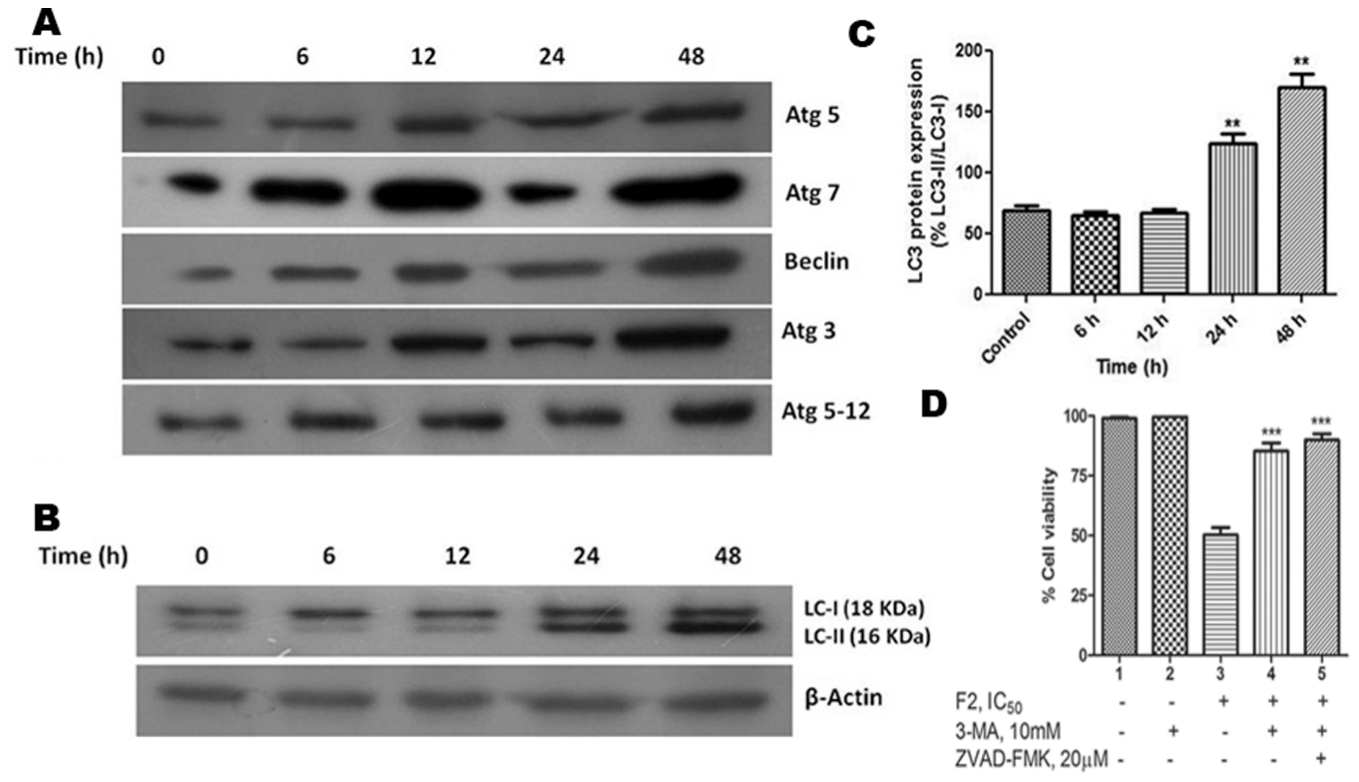
Expression of Autophagy proteins in U937 cells. (A) Cells were treated with F2 (18.6 µg/ml for 6 h, 12 h, 24 h, 48 h) and the expressions of Beclin-1, Atg 3, Atg 5, Atg 7, Atg 5-Atg 12 were analyzed by western blotting in whole cell extracts of control and treated cells. Analysis was confirmed with three different sets of experiments. β-actin served as a loading control. (B) Conversion of LC3-I to LC3-II. Whole cell lysates of control and F2 treated (18.6 µg/ml; 0–48 h) U937 cells were prepared and subjected to immunoblot analysis of expression level of LC3-I & LC3-II using anti LC-3 antibodies. β-actin was used to ensure equal loading. The figure is a representative profile of at least three experiments. (C) Densitometric study. Densitometric quantification of bands obtained by immunoblotting was done and LC3 protein expression was calculated as described in Materials and methods. Histograms represent LC3 protein expression normalized to β-actin (**p<0.01). (D) Contribution of autophagy in F2 induced cytotoxicity. Cells (2.5×10^4^/100 µl of RPMI 1640 medium/well) were pre-incubated with autophagy inhibitor 3-MA (10 mM) or both 3-MA (10 mM) and Z-VAD-FMK (20 µM) for 4 h before the addition of F2. Cell viability was evaluated by MTT assay after treatment with 0–100 µg/ml F2 for 48 h as described in Materials and methods. Histograms represent percentage cell viability (Mean ± SEM) obtained at the IC_50_ concentration of F2 (18.6 µg/ml) and has been derived from at least three experiments in duplicate (***p<0.001 compared to only F2 treated cells).

Bcl-2 protein has been mainly studied in the context of apoptotic cell death but there are other aspects to its function. Extensive studies have found that Bcl-2 binds to Beclin-1, a crucial protein in autophagosome formation; resulting in inhibition of autophagy [36]. Our results show a steady rise in Beclin-1 levels with increased duration of treatment (Fig. 7A). This is in concert with inhibition of Bcl-2 by F2. In order to affirm the occurrence of autophagy in F2 mediated death, expression of Atg proteins were studied. Atg proteins are a core set of proteins which are involved in a series of dynamic membrane-rearrangement reactions and tightly regulate all stages of autophagosome formation [37]. Hence we performed western blotting to analyze the changes in expression levels of Atg proteins. As shown in Fig. 7A, Atg 3, Atg 7, Atg 5, Atg 5-Atg 12 levels were markedly increased in a time-dependent manner after F2 treatment confirming the occurrence of autophagy.

### F2 Induced Conversion of LC3

It has been stated that under normal conditions, LC3-I is uniformly distributed throughout the nucleus and cytoplasm. During autophagy, LC3-I is processed into LC3-II and translocates into autophagosome membranes. Since the amount of LC3-II is clearly correlated with the number of autophagosomes, one approach to monitor autophagy is to detect LC3 conversion (LC3-I to LC3-II) [25], [38]. Hence, using Western blot analysis with anti-LC3 antibody, we examined the expressions of LC3-I (18 kDa) and LC3-II (16 kDa) in U937 cells after treatment with F2. As shown in Fig. 7B, an apparent increase in the levels of LC3-II protein was detected in U937 cells in a time dependent manner after treatment with F2. This was well subsidized by the densitometric data which demonstrated that level of LC3-II/LC3-I protein relative to β-Actin increased significantly after 24 h and 48 h of treatment (p<0.01) as compared to control (Fig. 7C). This suggests the induction of LC3-II, the form that is recruited to autophagosomes.

### F2 Induces Formation of AVO

**Figure 8:**
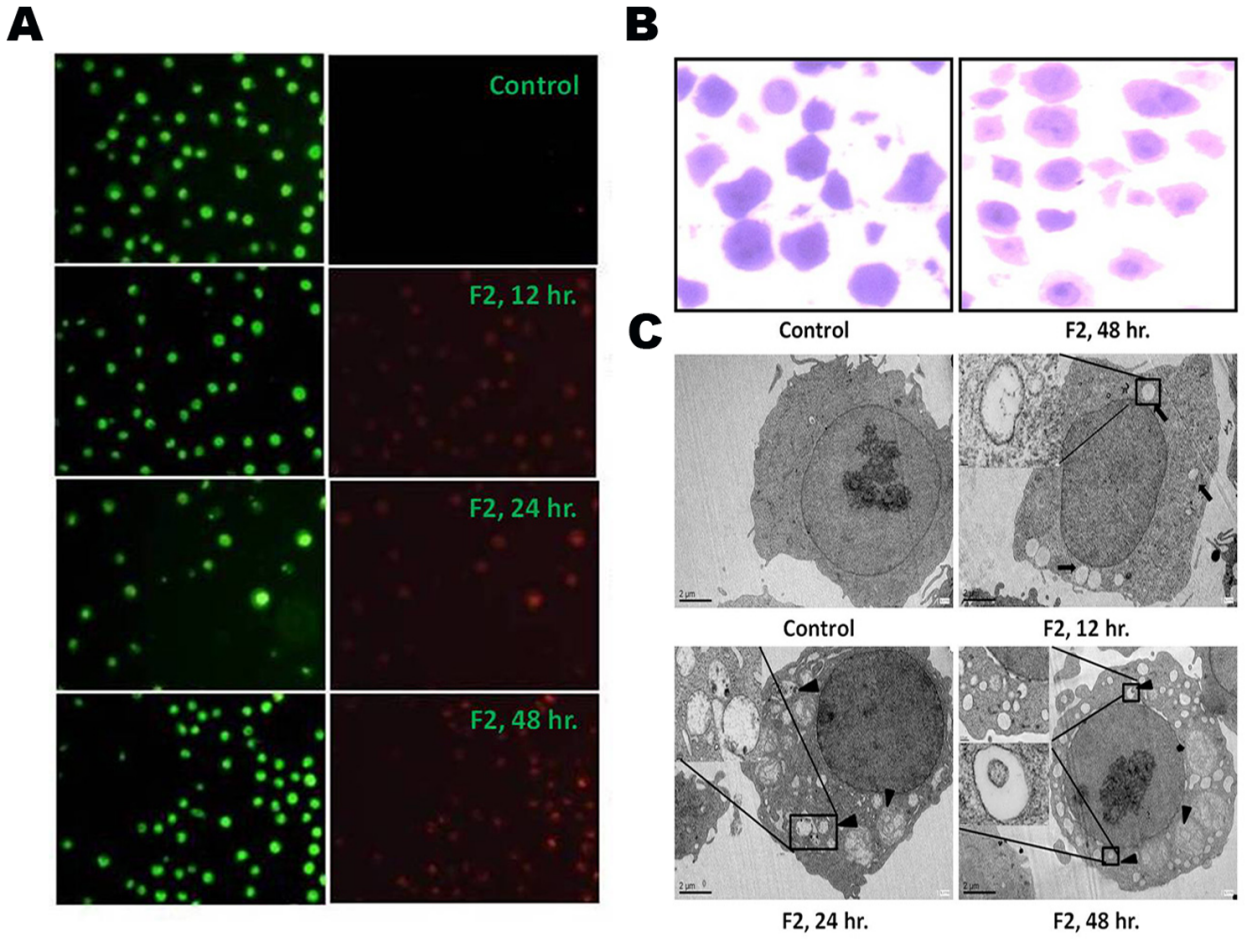
Induction of autophagy. (A) Formation of AVO. Control and F2 (18.6 µg/ml; 0–48 h) treated U937 cells (2.5×10^5^/ml) were stained with acridine orange (1 µg/ml) for 15 min and AVO formation observed under a fluorescence microscope (60X). At least 20 microscopic fields were observed for each sample. Formation of autophagosomes. (B) Control and F2 (18.6 µg/ml; 48 h) treated U937 cells were stained with toluidine blue and photographed under a light microscope (60X). At least 20 microscopic fields were observed for each sample. (C) TEM microphotographs showing the ultrastructure of control and F2 (18.6 µg/ml; 12 h, 24 h, 48 h) treated U937 cells. Double membraned autophagosomes (black arrow) and autolysosomes (black arrow heads) were observed in treated cells. The figure is a representative profile of at least three experiments.

A characteristic feature of cells engaged in autophagy is the formation of AVO [26], [39]. We visualized the effect of F2 on formation of AVO in U937 cells using fluorescence microscopy upon staining with the lysosomotropic agent AO. As shown in Fig. 8A, control cells primarily displayed green fluorescence with minimal red fluorescence, indicating a lack of AVO. On the other hand, U937 cells treated with F2 showed a time dependent increase in red fluorescent AVO, maximum being after 48 h of treatment. To testify the contribution of autophagy, cells were pre-treated with 3-MA (10 mM; 4 h), an inhibitor of autophagic sequestration and then incubated with F2 (0–100 µg/ml) for 48 h. 3-MA significantly attenuated F2 induced cytotoxicity in U937 cells (Fig. 7D) and IC_50_ increased to 50.0 µg/ml - reiterating the role of autophagy in F2 mediated cytotoxicity. Moreover, pretreatment of cells with both 3-MA and Z-VAD-FMK increased cell viability (Fig. 7D) and IC_50_ increased to 60.7 µg/ml which is not significantly higher than 50.0 µg/ml. This is consistent with the earlier mentioned fact that caspase does not solely decide the fate of F2 treated cells.

### F2 Induced Cell Death with Distinct Autophagic Morphology

Autophagy is featured by the formations of autophagosome and autolysosome [40]. By observation under a light microscope, U937 cells treated with F2 fraction (48 h) showed cytoplasmic vacuoles (Fig. 8B). This was confirmed using transmission electron microscopy, which demonstrated that control cells had a largely homogeneous cytoplasm while ultrastructure of treated cells showed the typical signs of autophagy. A number of isolated membranes, derived possibly from ribosome-free endoplasmic reticulum were observed. These membranes elongated and curved to engulf cytoplasmic fraction and organelles and eventually formed giant double membraned autophagosomes which fused with lysosomes resulting in the formation of autolysosomes. The formation of autophagosome was observed as early as 12 h of treatment and their fusion with lysosome began after 24 h of incubation with F2. Most of the autophagosomes contained lamellar structures or residual digested materials (Fig. 8C).

### F2 Induced Autophagy is ROS Dependent

**Figure 9:**
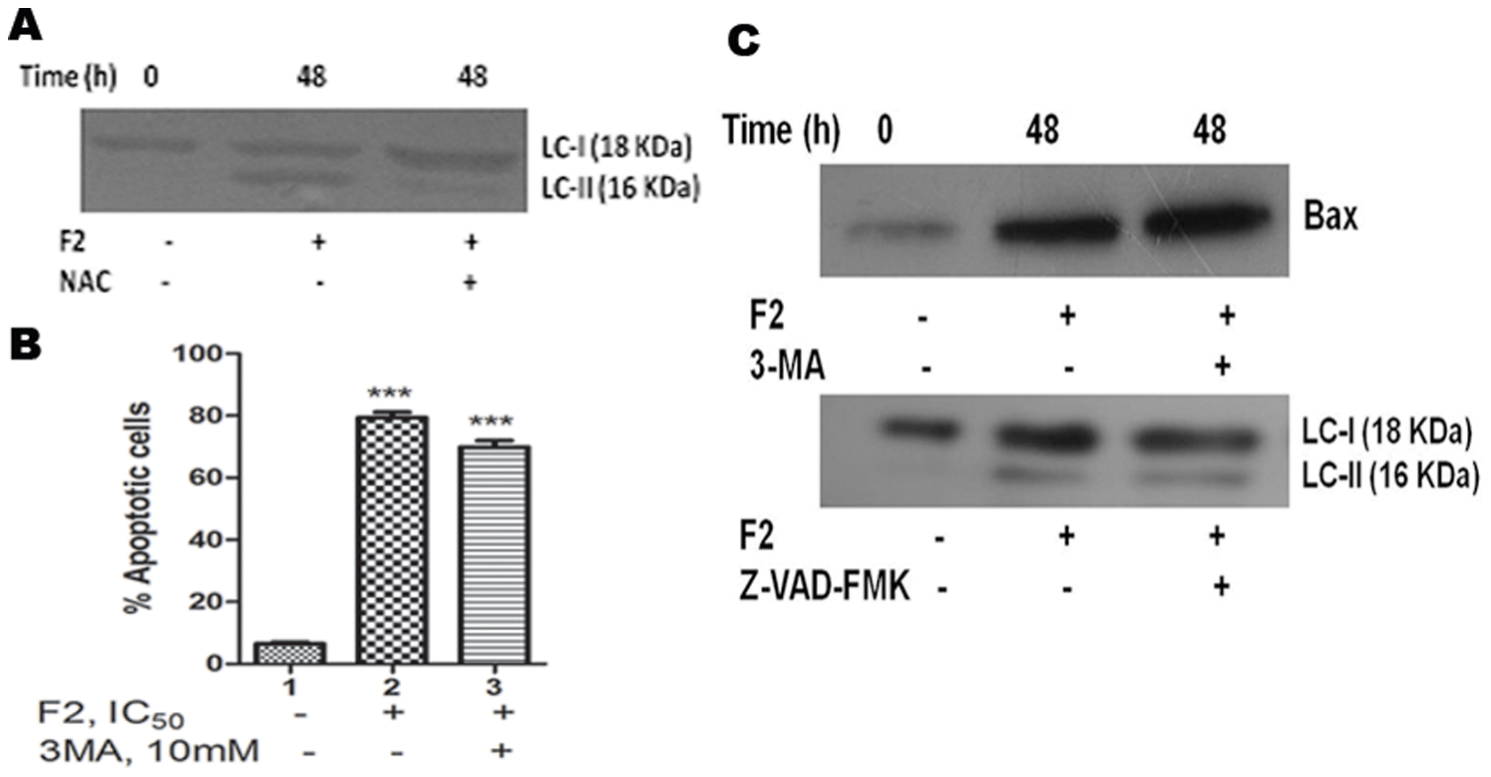
(A) Effect of anti-oxidant on F2 induced autophagy. Cells were treated with F2 (18.6 µg/ml; 48 h) with or without pre-incubation using NAC (2.5 mM; 3 h). Whole cell lysates were prepared and subjected to immunoblot analysis using specific antibodies against LC3. The figure is a representative profile of at least three experiments. (B) Effect of 3-MA on F2 induced Annexin V positivity. Cells were treated with F2 (18.6 µg/ml; 48 h) with or without pre-incubation using 3-MA (10 mM; 4 h). They were co-stained with PI and Annexin V-FITC followed by analysis using flow cytometry as described before. Histograms represent percentage of apoptotic cells and have been derived from at least three experiments (***p<0.001 compared to control cells). (C) Effect of 3-MA and Z-VAD-FMK on apoptosis and autophagy. Cells were treated with F2 (18.6 µg/ml; 48 h) with or without pre-incubation using 3-MA (10 mM; 4 h) or Z-VAD-FMK (20 µM; 4 h). Whole cell lysates were prepared and subjected to immunoblot analysis using specific antibodies against Bax or LC3. Analysis was confirmed with three different sets of experiments.

ROS mediated autophagy has been observed in a number of different cancer cells [41]. Having already identified that F2 induced generation of ROS, we next looked into the role of ROS in AG-4 induced autophagy in U937 cells. To investigate a functional link between ROS production and autophagy induction, we pre-incubated cells with NAC (2.5 mM; 3 h) followed by treatment with F2 (18.6 µg/ml; 48 h) and examined its effect on LC3 conversion. Attenuation of ROS levels by NAC significantly decreased the conversion of LC3-I to LC3-II upon F2 treatment as seen by immunoblotting methods (Fig. 9A). Thus, this suggests that F2 induced autophagy in U937 cells is dependent on ROS formation.

### Apoptosis and Autophagy Occur Independently upon F2 Treatment in U937 Cells

The results described thus far show that F2 induced both apoptosis and autophagy in U937 cells. The relationship between autophagy and apoptosis is complex and varies between cell types and the specific stress placed upon the cell. Both processes may be interconnected or may act independently [42]. To determine whether effects of F2 on apoptosis and autophagy are independent, or are a consequence of one process affecting the other, we analyzed cells treated with F2 in the presence of either apoptosis or autophagy inhibitors (Z-VAD-FMK and 3-MA respectively). Cells pre-incubated with 3-MA (10 mM; 4 h) followed by F2 (18.6 µg/ml; 48 h) treatment were subjected to western blot analysis and Annexin V assay. Fig. 9B revealed no appreciable fall in percentage of apoptotic cells in autophagy inhibited cells. Furthermore, Bax (a known apoptotic protein) levels increased to levels comparable to that of cells treated with only F2 (Fig. 9C). Similarly, lysates of cells pre-incubated with Z-VAD-FMK (20 µM; 4 h) followed by treatment with F2 were analyzed for conversion of LC3-I to LC3-II, molecular marker for autophagy. Fig. 9C suggests that LC3-I was processed to LC3-II as that of cells treated with only F2. These suggest that apoptosis and autophagy occur independently of each other in F2 induced cytotoxicity.

## Discussion

Contemporary therapeutic strategies solely relying on apoptosis have shown quite remarkable anticancer ability; nevertheless severe side-effects and chemoresistance have remained unavoidable. The search for new chemopreventive and antitumor agents that are more effective and less toxic has kindled great interest in phytochemicals. Even after chemotherapy most tumor cells retain the ability to sustain permanent growth arrest or undergo non-apoptotic types of cell death, such as necrosis, autophagy, senescence and mitotic catastrophe. A strategy that deliberately induces multiple mechanisms of cell death like apoptosis, autophagy could therefore be a useful adjunct to standard cancer therapies and might make a significant contribution to a favourable treatment outcome [43].

With a long history of clinical use in traditional medicine, SG and its roots, bark, leaves and flowers have been reported to have anti-inflammatory, analgesic, anti-pyretic and anti-epileptic effects. In this study, our objective was to unveil the molecular mechanism of cell death induced in human leukemic cells by a fraction derived from SG flowers. Since, normal proliferating cells have the same metabolic requirements as cancer cells, finding a therapeutic window between proliferating cancer cells and proliferating normal cells remains a major challenge in the development of successful cancer therapies [44]. Thus, development of compounds with the potential beneficial effect without cytotoxicity to normal cells has become the focal point of current research. In our study - F2, the most effective of all the fractions derived from SG flowers is able to selectively kill leukemic cells (U937, THP1, Raji and HL60) without much affecting solid tumor derived cells (MCF-7, HCT-15) and normal cells (NIH3T3 and normal human PBMC) (Table 2, Fig. 1). Importantly, F2 induced growth inhibitory effect in U937 cells demonstrated dose dependent as well as time dependent response (Fig. 1).

Apoptosis is a physiological process in normal cells and an invalid apoptosis pathway has often been one of the main hallmarks of cancer cells. Thereby, apoptosis induction by anticancer agents constitutes one aspect of treatment effect [45]. In our study, F2 caused externalization of phosphatidylserine (Fig. 2), indicating that F2 exerts its antiproliferative activity via apoptosis. Mitochondria are essential cellular organelles that play a central role in energy metabolism and are critical for the survival of any cell. Mitochondria may act as an orchestrator, integrating apoptotic and autophagic activity [46], making it as an attractive target for cancer chemotherapy. There is growing evidence, both in isolated mitochondria and in intact cells, illustrating many cancer chemotherapeutic agents modulate or interfere with mitochondrial functions to promote its dysfunction and depolarization. In mammalian cells, increased cellular ROS production has been suggested to be responsible for the depolarization of mitochondria and subsequent cell death. The rates of endogenous free radical generation and elimination are two important factors that determine the overall cellular ROS levels. As cancer cells have elevated ROS generation and are under increased intrinsic oxidative stress, it is conceivable that these malignant cells would be more vulnerable to further oxidative insults induced by ROS-generating agents [47], [21]. Exposure of U937, THP1, HL60, Raji cells to their respective IC_50_ concentration of F2, enhanced ROS generation; however it was not significantly higher compared to their untreated counterparts except in U937 cells. The generation of ROS was maximal in U937; however it does not impart significant stress to normal PBMC. F2 increased ROS levels in time-dependent and dose-dependent manners causing oxidative stress in U937 cells (Fig. 3A & B). Since ROS are chemically reactive, above basal levels they lead to cellular damage, including lipid peroxidation, oxidative DNA modifications, protein oxidation, and enzyme inactivation. The critical role of ROS was supported by the fact that anti-oxidant NAC completely attenuated the anti-proliferative and apoptotic effects of F2 (Fig. 3C & D). Furthermore, if ROS production is involved with the cytotoxic effect of F2, we would expect to observe a decline in cellular oxygen consumption since mitochondria consume ∼90% of cellular oxygen [48]. Indeed our results were consistent with earlier findings of depleted O_2_ consumption levels in U937 by F2 (Fig. 3E) which validates the crucial role of ROS in its cytotoxicity.

Inhibition of mitochondrial respiration has been demonstrated to favour mitochondrial depolarization. Furthermore, its dysfunction is a key event during apoptosis [49]. True to this fact, F2 induced generation of ROS was followed by disruption of the mitochondrial membrane potential in U937 cells (Fig. 4A). Given the importance of mitochondria, its dysfunction brings the cells to a “point of no return” [50]. Apoptotic stimuli, regulated by the opposing actions of pro- and anti-apoptotic members of the Bcl-2 family, induce the opening of membrane permeability transition pores and swelling of mitochondrial matrix [50], [51]. Increased levels of Bax proteins have been reported to directly induce the release of cytochrome c by forming a pore in the outer membrane of the mitochondria. In contrast, Bcl-2 preserves mitochondrial integrity and through the finetuning of intracellular levels of ROS, maintains intracellular redox status at a level optimal for cell survival [50]. A recent report has also stated that overexpression of Bcl-2 increases oxygen consumption and in doing so blocks death signaling [52]. Consistent with enhanced ROS generation, inhibited oxygen consumption and depolarization of mitochondria, we observed Bax activation and depleted levels of Bcl-2 (Fig. 4B). Furthermore, prominent anti-apoptotic Bcl-2 family members e.g. Bcl-2, were originally identified and found to be overexpressed in leukemic cells [53], [54]. Therefore, the ability of F2 to induce downregulation of Bcl-2 in U937 cells holds great significance. Consequently, altered expression levels of Bax and Bcl-2 led to the release of cytochrome c (Fig. 4C); indicating possible mitochondrial apoptosis in F2 treated U937 cells. All these events point to the occurrence of mitochondrial dysfunction as a key event in F2 fraction treated cells.

Caspase-mediated cleavage of specific substrates explains several of the characteristic features of programmed cell death viz. chromatin condensation, nuclear shrinkage, DNA fragmentation [23]. The released cytochrome c promotes the activation of caspase cascade leading to cleavage of the DNA repair enzyme, PARP eventually causing DNA degradation [55]. The cleavage of PARP protein can therefore be considered as a molecular marker for caspase activation. In our study, F2 fraction increased the activity of caspases-8, -9, and -3 (Fig. 5A) and the presence of cleaved PARP in F2 treated U937 cells (Fig. 5B) which confirms caspase activation. However, the fate of the treated cells is not solely determined by the caspase-mediated pathway. Only Z-VAD-FMK failed to retrieve the viability of F2 treated cells (Fig. 5C). Also the percentage of apoptotic cells showed no substantial decline when cells were pre-incubated with Z-VAD-FMK followed by treatment with F2 (Fig. 5D). This suggests the presence of an alternate pathway which led us to investigate the expression of AIF which when translocated to nucleus mediates a caspase-independent process. As envisaged, F2 fraction caused a time-dependent increase in AIF levels in nuclear protein extract of U937 cells (Fig. 5E) – evidence enough for its translocation to nucleus. Both- caspases and AIF are known to be involved in DNA fragmentation [23], [35]. True to this, F2 caused DNA fragmentation as observed by fragmented nuclei in Hoechst 33258 stained cells (Fig. 6). Moreover, cells were unable to repair the fragmented DNA due to impairment of PARP leading to cellular demise.

Altered expression of Bcl-2 may not only be a mechanism for regulating apoptosis but also indirectly modulates autophagy [56]. Autophagy is a genetically programmed, evolutionarily conserved physiological process by which cytoplasmic materials including damaged proteins and organelles are sequestered for lysosome-dependent degradation. It is a dynamic process consisting of several consecutive stages involving the following sequential steps: (a) induction or initiation, which involves formation of phagophores (b) nucleation; (c) elongation - a critical step in forming the complete double membraned autophagosomes (d) maturation and degradation, which involves fusion with endosomes-lysosomes to form autophagolysosomes and degradation of the inner membrane together with its luminal contents [57], [58], [59]. Beclin-1, a BH3- (Bcl-2-homology-3-) only protein, is a part of type III PI3 kinase complex and plays a crucial role in the nucleation stage of autophagosome formation. It was initially discovered as a Bcl-2-interacting protein [60] and was also identified as a tumor suppressor in various types of cancer [61], [62], [63], indicating its important role in autophagic cell death during cancer therapy. Bcl-2 functions as an anti-autophagy protein via its inhibitory interaction with Beclin-1 [64]. F2 promoted beclin-1 levels in U937 cells (Fig. 7A) and this is consistent with depletion in Bcl-2 expression. Two ubiquitin-like conjugation pathways are involved in the vesicle elongation process. One pathway involves the covalent conjugation of Atg 12 to Atg 5 with the help of Atg 7. F2 fraction increased expression of Atg 7, Atg 5 and the conjugated form Atg 5-Atg 12 (Fig. 7A) implying the presence of this pathway. The second pathway involves the conjugation of phosphatidylethanolamine to LC3 in which Atg 3 plays a crucial role. Lipid conjugation converts cytoplasmic LC3 (LC3-I) to a double layer autophagic membrane-associated form (LC3-II) [26], [65]. F2 was able to enhance levels of Atg 3 and conversion of LC3-I to LC3-II (Fig. 7A, B & C), evidence for the existence of the second pathway in F2 treated cells. The vacuoles (autophagosomes) undergo acidification after maturation. Autophagosomes then fuse with lysosomes (called autophagolysosome or AVO), where their materials are sequestered for degradation [66]. These are detected by supravital stain AO which emits red fluorescence under acidic conditions. Cells treated with F2 and stained with AO demonstrated a time dependent increase in red fluorescent structures in U937 cells, evidence of AVO formation (Fig. 8A). Classically, electron microscopy has been considered as the gold standard to demonstrate autophagic morphology in cells [40]. Transmission electron microscope figures serve as evidence of the formation of autophagosomes and autophagolysosomes after treatment with F2 fraction (Fig. 8C). In totality, all these point to the fact that F2 contributes to all steps of autophagosome formation and its biochemical signature, thus it can be regarded as a potent inducer of autophagy. This leads to disruption of cytoplasm organelles [67]. Autophagy is thought to contribute to cell survival as well as cellular demise and this is purely circumstantial [68]. To determine the contribution of autophagy in F2 fraction induced death of U937 cells, 3-MA (commonly used as a specific inhibitor of autophagic sequestration) was used. Interestingly, 3-MA was shown to confer protection against F2 induced cytotoxicity (Fig. 7D). In addition, Z-VAD-FMK along with 3-MA also reversed the cytotoxic effect of F2. This implied that autophagy plays an important role in F2 fraction induced death in U937 cells. Since inhibition of autophagy significantly affects the anti-proliferative effect of F2 fraction it serves as a cell death mechanism in U937 cells after treatment with F2 fraction. Besides its role in apoptosis, there is accumulating evidence for selective autophagic processes in response to ROS. Pre-treatment with NAC diminished expression levels of LC3-II – signifying ROS mediated autophagy after F2 treatment (Fig. 9A). The functional relationship between apoptosis and autophagy is obscure and circumstantial; in some cases apoptosis and autophagy act antagonistically while in other cases autophagy enables apoptosis [65]. Since, F2 was able to induce both apoptosis and autophagy in U937 cells; we investigated for apoptosis and autophagy in presence of 3-MA and Z-VAD-FMK respectively. F2 treated cells showed increased Bax levels and high percentage of apoptotic cells even in presence of 3-MA and conversion of LC3-I to LC3-II in presence of Z-VAD-FMK (Fig. 9B & C). This implied independent occurrence of apoptosis and autophagy after F2 treatment.

In summary, our study provides evidence that F2, a fraction isolated from SG flowers, preferentially kills leukemic cells (particularly those of histiocyte lymphoma) by triggering programmed cell death. F2 induced cytotoxicity involved phosphatidylserine exposure, enhanced ROS generation leading to altered mitochondrial bioenergetics and apoptotic protein expressions. This culminated in caspase activation and DNA fragmentation. Even with these molecular features of apoptosis, F2 was able to induce autophagy as evidenced by autophagosome formation, LC3 conversion and altered Atg protein expressions. Apoptosis and autophagy occurred independent of each other in the context of F2 induced death. To the best of our knowledge, this study is the first to demonstrate that fraction F2 derived from SG flowers induces autophagy. Thus, these findings add a new avenue to SG induced cell death pathways and offers significant promise for future cancer therapeutics. Further studies are underway to isolate active compounds from this fraction and to explore signal transduction pathways regulating programmed cell death induced by the active compounds in quest of an interesting formulation which can be used in the treatment of cancer.
